# Integrin β1 Optimizes Diabetogenic T Cell Migration and Function in the Pancreas

**DOI:** 10.3389/fimmu.2018.01156

**Published:** 2018-05-31

**Authors:** Gabriel Espinosa-Carrasco, Cécile Le Saout, Pierre Fontanaud, Aurélien Michau, Patrice Mollard, Javier Hernandez, Marie Schaeffer

**Affiliations:** ^1^INSERM U1183, Institute for Regenerative Medicine and Biotherapy, University of Montpellier, Montpellier, France; ^2^Institute of Functional Genomics, University of Montpellier, CNRS, INSERM, Montpellier, France

**Keywords:** autoimmunity, T cell migration, type 1 diabetes, imaging, *in vivo*

## Abstract

T cell search behavior is dictated by their need to encounter their specific antigen to eliminate target cells. However, mechanisms controlling effector T cell motility are highly tissue-dependent. Specifically, how diabetogenic T cells encounter their target beta cells in dispersed islets throughout the pancreas (PA) during autoimmune diabetes remains unclear. Using intra-vital 2-photon microscopy in a mouse model of diabetes, we found that CXCR3 chemokine downregulated CD8^+^ T cell motility specifically within islets, promoting effector cell confinement to their target sites. By contrast, T cell velocity and directionality in the exocrine tissue were enhanced along blood vessels and extracellular matrix fibers. This guided migration implicated integrin-dependent interactions, since integrin blockade impaired exocrine T cell motility. In addition, integrin β1 blockade decreased CD4^+^ T cell effector phenotype specifically in the PA. Thus, we unveil an important role for integrins in the PA during autoimmune diabetes that may have important implications for the design of new therapies.

## Introduction

Immune responses implicate sequential encounters between T cells and their specific (or cognate) antigen in different body compartments to ensure efficient T cell priming, activation, and antigen clearance (1, 2). However, the frequency of naïve T cells specific for a given antigen is low (3), and antigen abundance in target tissues may be variable and/or spatially restricted. Thus, T cell search behavior is driven by the need to actively explore the environment and locate cognate antigens. Since T cell migration patterns depend on cell-intrinsic parameters, context-dependent micro-environmental chemotactic cues and tissue-dependent structural features (4, 5), empirical studies are required to identify T cell search mechanisms in specific disease settings. Given the importance of T cell search strategies in target cell clearance (1, 2), mechanisms involved may constitute promising new therapeutic targets.

Dynamics and mechanisms of T cell migration leading to initial antigen encounter in secondary lymphoid organs are best characterized (4, 6, 7). In lymph nodes (LNs), the frequency of naïve antigen-specific T cells is low (3) and migration patterns must optimize the likelihood of a productive encounter with a cognate antigen-bearing antigen-presenting cell (APC). Hence, naïve T cells typically display a high velocity dependent on chemokines and interactions with dendritic cells (8, 9). They migrate following a “Brownian” random walk intrinsically encoded (7, 10) and guided by a network of fibroblast reticular cells (11). This ensures efficient sampling of a multitude of APCs (6) to promote rare cognate antigen encounter and naïve T cell activation. Activated effector T cells with reprogrammed expression of adhesion molecules and chemokine receptors then migrate to peripheral tissues (12), where they usually accumulate in large numbers and need to search for their spatially restricted cognate antigen (3), to maintain effector functions (13) and eliminate target cells (14).

While the unique LN architecture facilitates antigen-T cell encounters, peripheral tissue geometry and composition greatly impact T cell migratory patterns and speed (1, 10, 15–17). For instance, vascular network, APC networks, and the extracellular matrix (ECM) architecture influence T cell interstitial trafficking through physical or/and adhesive guidance (15, 17–19). While adhesion-dependent mechanisms are not required for interstitial migration and T cell motility in LNs is integrin-independent (20, 21), T cells are able to switch migration modes *in vitro* (22) and inflammation-mediated changes in ECM composition in peripheral tissues are able to induce integrin-dependent T cell trafficking (1). Thus, predicting disease-dependent mechanisms controlling T cell motility in the periphery remains impossible, although these may play a crucial role in target cell clearance (1, 2).

During type 1 diabetes (T1D), an autoimmune disease leading to the destruction of insulin-producing pancreatic beta cells, T cells become activated in the draining LNs (23). Effector T cells then migrate to the pancreas (PA) and extravasate both within islets (24) and at post-capillary venules in the exocrine tissue (14). Furthermore, effector T cells have been shown to displace from one islet to another (14). These observations indicate that the migration of T cells in the exocrine tissue to reach dispersed target islets is essential for disease progression. However, mechanisms governing their motility remain unclear. Recent work in a viral-induced mouse model of diabetes described diabetogenic T cell motility as a Brownian-type random walk around islets (14), whereas in NOD mice, they appear to migrate along blood vessels (19). Given the extensive ECM remodeling and the key role of ECM organization in T1D pathogenesis (25), we sought to investigate mechanisms of effector T cell interstitial migration in the PA during T1D onset, using intra-vital 2-photon imaging in a mouse model of autoimmune diabetes.

## Materials and Methods

### Ethical Statement

Animal studies were conducted according to the European guidelines for animal welfare (2010/63/EU). Protocols were approved by the Institutional Animal Care and Use Committee (CEEA-LR-1190 and -12163) and the French Ministry of Agriculture (APAFIS#3874).

### Mice

Mice were bred in a specific pathogen-free facility and housed in conventional facility during experimentation. The transgenic mouse model of diabetes (26, 27) involved InsHA (28), Clone 4 TCR (MHC class I-restricted) (29), and HNT TCR (MHC class II-restricted) mice (30) (from Prof. Sherman, The Scripps Research Institute, San Diego, CA, USA) (27), RIPmCherry mice (31) (from the National Institute of Medical Research, London, UK), and β-actin-GFP and -CFP mice (Jackson Laboratory). Clone 4 TCR Thy1.1 x β-actin-GFP, HNT TCR Thy1.1 x β-actin-CFP, and InsHA x RIPmCherry mice on BALB/c x C57BL/6 background 10–16 weeks old were used (27). Littermate males and females were used whenever possible and homogeneously mixed between experimental groups.

### T Cell Isolation, Adoptive Transfer, and Diabetes Monitoring

Equal numbers (2–3 × 10^6^ cells/recipient) of naïve CD8^+^ and CD4^+^ T cells isolated from Clone 4 TCR Thy1.1 x β-actin-GFP and HNT TCR Thy1.1 x β-actin-CFP mice, respectively, were injected i.v. into InsHA x RIPmCherry mice sub-lethally irradiated (4.5 Gy) 24 h before in a therapeutic irradiator (Varian), as described (27). Mice were used for intra-vital imaging, sacrificed at day 10 for T cell characterization or monitored for diabetes onset. Recipient mice blood glucose levels were measured using a glucometer (AccuCheck).

### *In Vivo* Antibody and Peptide Treatment

To determine optimal imaging time post injection and control for potential micro-anatomical changes between different imaging fields, we first injected mAbs through a catheter inserted in the jugular vein and monitored average T cell motility in the same field pre- and post injection. A maximum effect was obtained 35–50 min post injection using blocking mAb, and isotype control antibodies had no effect on T cell motility. This is in agreement with our previous observations (27). Thus, blocking mAb or isotype control antibodies were injected i.v. on day 8 after T cell transfer 1 h prior to imaging, and blocking mAb-treated animals were directly compared to isotype control antibody-treated animals. Anti-CXCR3 (armenian hamster IgG, BioXcell) and isotype control polyclonal armenian hamster IgG (BioXcell) were used at 300 μg/mouse, and anti-β_1_ integrin (Hmβ1-1, eBioscience) and isotype control armenian hamster IgG H4/8 (eBioscience) were used at 100 μg/mouse. GRGDS peptide or control reverse SDGRG peptide (Sigma) (500 μg/mouse) were injected i.v. 10 min prior to imaging. For characterization of donor T cells by FACS, anti-β_1_ integrin or isotype control antibody was injected i.p. on days 8 (200 μg/mouse) and 9 (100 μg/mouse) and mice sacrificed at day 10 after T cell transfer.

### Surgery and Intra-Vital Imaging

All experiments used normoglycemic mice. Animals were anesthetized by the injection of ketamine/xylazine (0.1/0.02 mg/g). PA was exteriorized by surgery as described (27, 31). Fluorescence was visualized using a Zeiss 7MP 2-photon microscope adapted with an M Plan Apo NIR × 20 objective (0.4 NA, Mitutoyo). Excitation was achieved using a Ti:Sapphire Chameleon Laser (Coherent) tuned to either 820 nm [mCherry, mCherry-GFP-CFP excitation and second harmonic generation (SHG)], 850 nm (rhodamine-GFP-CFP), or 880 nm (GFP-CFP). Fluorescence was captured using GaAsP PMTs at 460–500 nm for CFP, 500–550 nm for GFP, 610–700 nm for mCherry and rhodamine, and <410 nm for SHG. Surface islets (<100 µm) were identified using mCherry or by light contrast. Tissue viability was verified by fluorescent dextran injection i.v. (27).

### Image Data Analysis

Stacks 150–250 µm thick (Z steps of 3 µm) were acquired every 30 s to 1 min for 10–27 min. Movies were stabilized using Huygens Essential (SVI). Measurements were performed in at least three independent experiments. Average velocities and mean squared displacements (MSD) of individual T cells were obtained using Imaris (Bitplane). Directionality indexes (ratio between the distance between start and end time points in a straight line and the total length of the migratory path) were calculated using a routine programmed in MATLAB (18). Similarly, T cell coordinates obtained using Imaris were imported in MATLAB to measure the displacement of T cells toward or away from islet centroids, to project T cell orientation of displacement vectors on a circle, to calculate angle differences between T cell displacement vector projections on the *XY* plane and the direction of vessels, and to generate graphs of *XY* projections of T cell tracks, using custom programs (available upon demand). Randomly selected areas with similar infiltration (100–320 total number of tracks/0.05 mm^3^ imaging volume) were compared between the different groups. Cell tracks lasting less than 4 min were excluded. No exclusion was made based on velocity.

To analyze migratory patterns of T cell populations, equations describing the major models of diffusion of particles (*Y* = *B*1 × *t* + (*B*2 × *t*)^α^) with α = 2 for directed or ballistic motion, α = 1 and *B*2 = 0 for Brownian random walk, 0 < α < 1 and *B*1 = 0 for sub-diffusive or anomalous random walk, 1 < α < 2 and *B*1 = 0 for Lévy-type super-diffusive random walk, and *Y* = Plateau × [1-exp(-*K* × *t*) for confined motility] (32) applied to the description of T cells migration (4) were used as models of nonlinear regression to fit MSD increase over time in GraphPad Prism (*t* is time, *B*1 and *B*2 are fitting parameters, *K* is the constant rate). In each case, the model providing the best fit (highest *R*^2^) was chosen to describe the pattern of motility.

### Flow Cytometry

For T cell phenotyping, single cell suspensions from pancreatic LNs or PA infiltrating cells were prepared and stained as described (26). For intracellular cytokine staining, T cells were restimulated *ex vivo* with hemagglutinin (HA)-specific peptides during 5 h before staining as previously described (26). The mAbs used were anti-CD61 (ITGβ3)-FITC, anti-CD51 (ITGαV)-PE, anti-CD49e (ITGα5)-APC, anti-CD183 (CXCR3)-Alexa Fluor 780, anti-CD29 (ITGβ1)-Pacific blue (BioLegend, San Diego, CA, USA); anti-CD4-V500, anti-CD4-FITC, anti-CD90.1 (Thy1.1)-PerCP, anti-CD90.1 (Thy1.1)-V450, anti-CD8a-V450, anti-CD62L-APC, anti-IL-2-APC, anti-IFNγ-PE (BD Pharmingen); anti-CD8a-APC-Alexa Fluor 780, anti-CD25-APC-Alexa Fluor 780, and anti-KLRG1-PE-Cy7 (eBioscience). Cells were analyzed on a FACSCanto II or a LSR Fortessa apparatus using Diva software (BDB).

### Confocal Imaging

Pancreas preparation and antibody labeling were as described (33). Antibodies used were as follows: hamster anti-CD11c (clone N418 1:300, eBioscience); rat anti-F4/80 (clone MCA4976 1:200, BioRad), rabbit anti-insulin (1:500, Cell Signaling); rat anti-endomucin (1:500, Santa Cruz Biotechnology); rabbit anti-fibronectin (clone AB1942 1:5,000, Chemicon); and mouse anti-collagen I (1:300, Abcam). Nuclei were labeled using dapi (Sigma). One to four slices were randomly selected from >3 animals/groups. Images were acquired using a Zeiss LSM 780 confocal microscope and analyzed using Imaris (Bitplane) and ImageJ (NIH).

### Statistical Analysis

Values are represented as mean ± SEM. Statistical tests were performed using GraphPad Prism. Normality was tested using D’Agostino–Pearson test, and comparisons were made using either unpaired Student’s *t*-test, or two-tailed Mann–Whitney *U*-test, as appropriate. Multiple comparisons were made using one-way ANOVA followed by Bonferroni’s *post hoc* test. To analyze uniformity of distribution, the Hodjes–Ajne test for circular uniformity was used in MATLAB. *P*-values were considered significant at *P* < 0.05*, 0.01**, 0.001***, and 0.0001****.

## Results

### Effector T Cells Follow a Lévy Walk Type of Motility in the Exocrine Tissue

**Figure 1:**
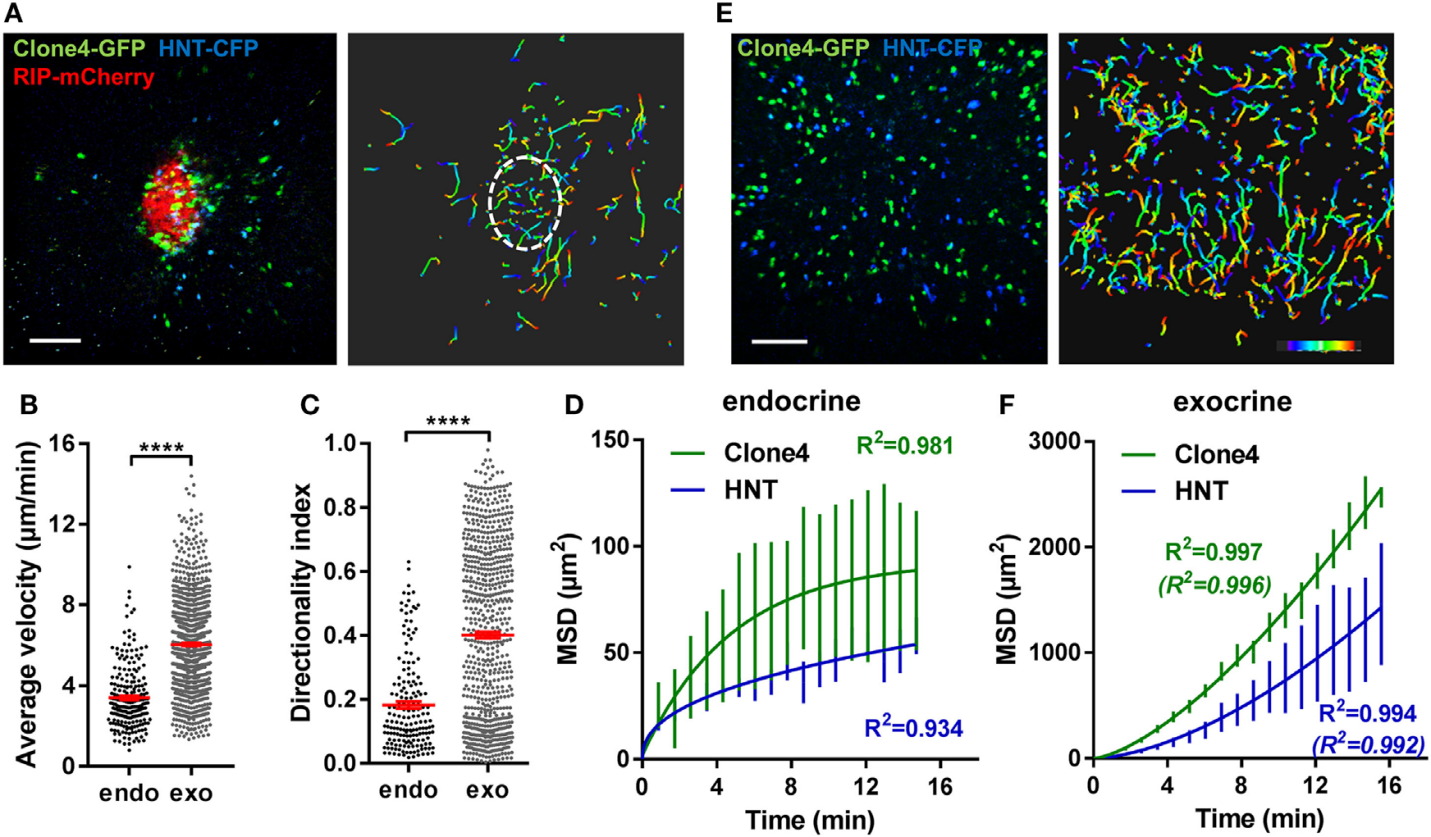
Motility of islet-antigen-specific CD8^+^ and CD4^+^ T lymphocytes *in vivo*. Irradiated InsHA-mCherry mice adoptively transferred with Clone 4-GFP CD8^+^ and HNT-CFP CD4^+^ T cells were subjected to intra-vital microscopy on day 8. **(A)** Still image from a representative movie (left panel; scale: 100- and 200-µm *Z*-projection; red: mCherry, green: GFP, blue: CFP) (see Video S1 in Supplementary Material) and the corresponding T cell tracks (right panel), color-coded as a function of time. Islet is circled. Movie duration: 15 min. **(B)** Average velocities of pooled CD4^+^ and CD8^+^ T cells in exocrine and endocrine tissues (*n* = 4 mice/condition; 1–2 movies/mouse, Mann–Whitney). Dots correspond to individual T cells. **(C)** Directionality indexes (ratio between displacement and total track length) of T cells in exocrine and endocrine tissues (*n* = 4 mice/condition; 1–2 movies/mouse, Mann–Whitney). Dots correspond to individual T cells. **(D)** Mean squared displacement (MSD) of T cells as a function of time in islets, best fitted with a confined model of migration for Clone4-GFP cells and with sub-diffusive random walk for HNT-CFP cells. Bars correspond to SEM (*n* = 4 mice/condition; 1–2 movies/mouse). **(E)** Still image from a representative movie in the exocrine tissue (left panel; scale: 100- and 200-µm *Z*-projection; green: GFP, blue: CFP) (see Video S2 in Supplementary Material) and the corresponding T cell tracks (right panel), color-coded as a function of time. Movie duration: 19 min. **(F)** MSD of T cells as a function of time in the exocrine tissue, best fitted with a Lévy walk model of migration. Between brackets are *R*^2^-values of fit for ballistic (directed) motility. Bars correspond to SEM (*n* = 4 mice/condition; 1–2 movies/mouse).

To study antigen-specific T cell behavior and motility patterns in the PA during autoimmune diabetes, we used the InsHA transgenic mouse model (34) in which fluorescent labels were introduced (27). We imaged influenza HA antigen-specific CD8^+^ and CD4^+^ T cells attacking HA-expressing beta cells, utilizing *in vivo* 2-photon microscopy (27, 31). Co-transfer of naïve Clone 4-GFP CD8^+^ and HNT-CFP CD4^+^ T cells into sub-lethally irradiated InsHA-mCherry hosts reproducibly induced PA infiltration by day 8 post transfer (Figure S1A in Supplementary Material) and hyperglycemia by day 10 (Figure S1B in Supplementary Material). We were able to image beta cells, Clone 4-GFP CD8^+^ and HNT-CFP CD4^+^ T cells in pre-diabetic InsHA-mCherry mice and track T cell motility *in vivo* (Figure 1A; Video S1 in Supplementary Material). At day 8 post transfer, HA-specific T cells in endocrine tissue (in islets) displayed lower average velocities than in the surrounding exocrine tissue (27) and low directionality indexes (<0.2) (ratio between cell’s displacement, defined as the straight line between the original and the final positions, and the cell’s total track length) (Figures 1B,C), as expected for T cells in the presence of their cognate antigen (35). To describe T cell migration patterns, particle diffusion models have classically been used (32). T cells mostly migrate either following a Brownian-type random walk or a super-diffusive Lévy-type motility (characterized by stretches of directed motility in random directions interleaved by pauses) (4). Occasionally, T cells can display restrained motility (anomalous random walk or confinement) (8) or fully ballistic migration (in a straight path) (36), depending on the imaging duration and the tissue analyzed. These models are based on the representation of cells’ MSD versus time (4). We fitted the experimental data with the different equations describing different models of diffusion (32) and identified the best fit based on the *R*^2^-values. While a complete Brownian-type random walk yields a linear regression between these parameters, a directed motility or a super-diffusive motility typical of a Lévy walk is characterized by a power law curve, and confinement leading to sub-diffusive behavior yields a hyperbolic-shaped curve. Analyses of MSD of T cell populations versus time in islets revealed that CD8^+^ T cell migration was best fitted with a model of confined motility, while CD4^+^ T cells migrated following a sub-diffusive (also called anomalous or restrained) random walk (Figure 1D). In the exocrine tissue, the mean T cell directionality index was in the 0.4 range (Figure 1C), consistent with values reported for CTLs in a different model of insulitis (14) and indicative of an apparent lack of directionality. However, Clone 4-GFP and HNT-CFP T cell motility in the exocrine PA of InsHA-mCherry mice did not follow the described Brownian-type strictly diffusive random motility (14), and MSD of both T cell populations versus time (4) was best fitted with a model of super-diffusive Lévy-type motility, closely tending to a directed ballistic migration (36) (Figures 1E,F; Video S2 in Supplementary Material).

### Contribution of Chemotactic Cues to T Cell Exploratory Migration in the PA

**Figure 2:**
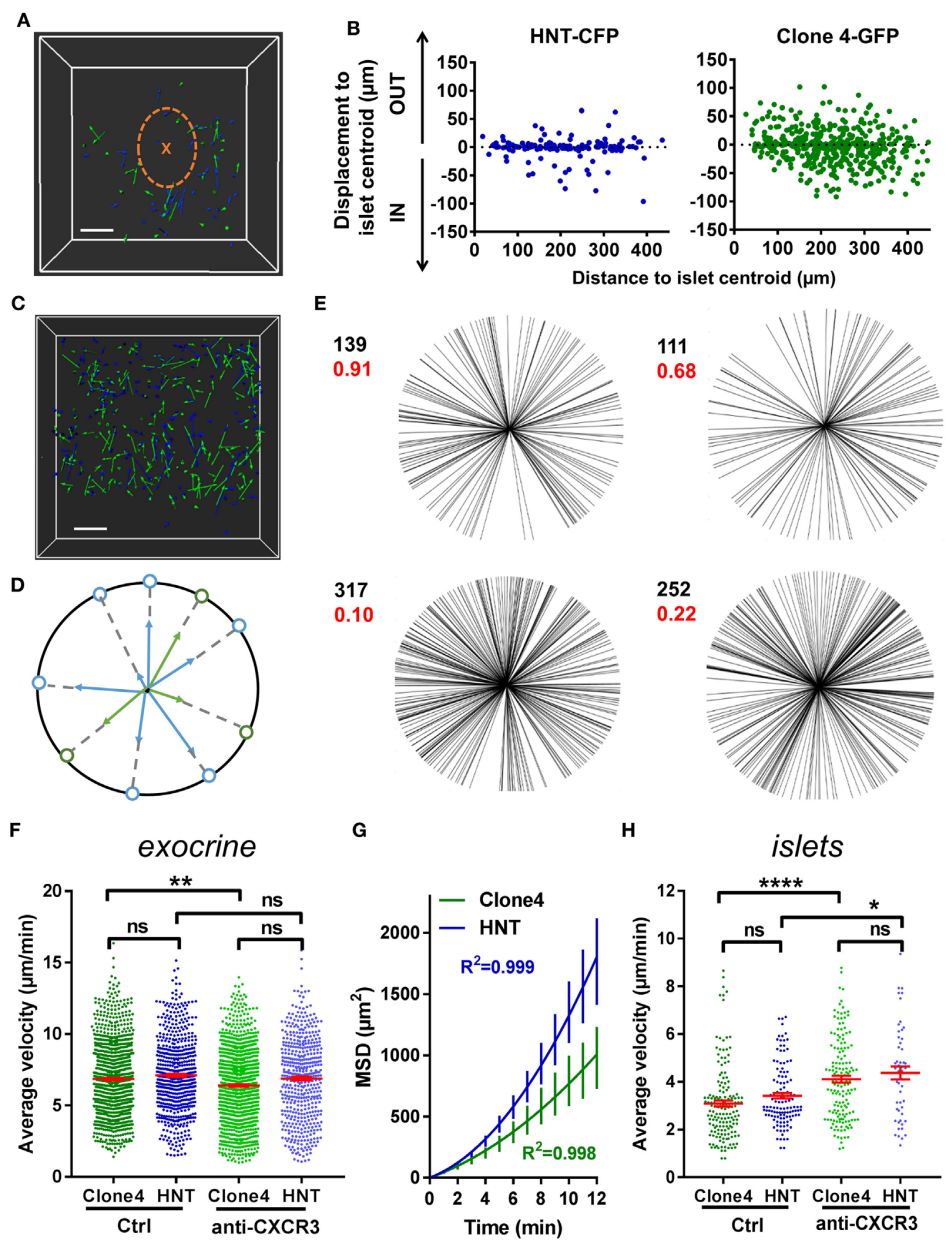
T cells collective migration is not biased toward islets and is mostly independent of CXCR3 signaling. Irradiated InsHA-mCherry mice adoptively transferred with Clone 4-GFP CD8^+^ and HNT-CFP CD4^+^ T cells were subjected to intra-vital microscopy on day 8. **(A)** *XY* projections of track displacement vectors of T cells in movie in Figure 1A (see Video S1 in Supplementary Material). Scale: 100 µm. Blue and green tracks correspond to HNT-CFP and Clone 4-GFP T cells, respectively. Islet is circled, and a cross marks islet centroid. **(B)** Clone 4-GFP and HNT-CFP displacement during movies toward (IN) or away (OUT) from islets, as a function of distance from islet centroid at the start of movies (*n* = 4 mice; 1–2 movies/mouse). **(C)** *XY* projections of T cell track displacement vectors in exocrine tissue (see Video S2 in Supplementary Material) (scale: 100- and 200-µm *Z*-projection; green: GFP, blue: CFP). Movie duration: 10 min. **(D)** To analyze orientations of T cell directions, displacement vectors were projected on the *XY* plane and set to a common origin. The orientation of each vector was projected on a circle. **(E)** Statistical analysis of T cell track orientations in four different movies (without islet) (numbers in black correspond to the number of tracks). None of the analyzed distributions were significantly different from a uniform distribution (Hodjes–Ajne test for circular uniformity, *P*-values in red). **(F)** The average velocities of CD4^+^ and CD8^+^ T cells in the exocrine tissue (*n* = 4–6 mice/condition; 2–3 movies/mouse, one-way ANOVA). Dots correspond to individual T cells. CXCR3: anti-CXCR3 mAb-treated mice. **(G)** MSD of T cells as a function of time in the exocrine tissue, best fitted with a Lévy walk super-diffusive model of migration. Bars correspond to SEM (*n* = 4 mice per condition; 1–2 movies/mouse). **(H)** The average velocities of CD4^+^ and CD8^+^ T cells in islets (*n* = 5–6 mice/condition; 1 movie/mouse, one-way ANOVA). Dots correspond to individual T cells. Bars correspond to SEM.

Chemotaxis refers to the capacity of T cells to adapt their migratory pattern and motility following sensing of extrinsic cues produced by other immune cells or tissue-specific cells. To analyze whether the super-diffusive motility in the exocrine tissue was informed by chemotactic cues produced within infiltrated islets, which are important sources of chemokines (37), and whether T cells were able to collectively migrate toward islets, we analyzed displacement of T cells toward (IN) or away (OUT) from islet centroids, as a function of T cell initial position (Figures 2A,B). Proximity to islets did not bias T cell orientation of movement, as described in another model (14). Furthermore, although T cells migrated in rather straight paths in the exocrine tissue, cells did not collectively migrate in one particular direction in movies, as the distribution of compiled T cell vector orientations in different movies did not statistically differ from a uniform circular distribution (Figures 2C–E). Thus, T cells do not seem to collectively respond to a large-scale chemoattractive gradient.

An alternative possibility may be that T cells are able to respond to chemotactic cues locally (24). While T cells search for their cognate antigen, APCs are able to recruit them through secretion of different chemokines (38–40). CXCL10 is the most abundant chemokine expressed in infiltrated PA in mouse models, including InsHA, as well as in type 1 diabetic patients, and this chemokine contributes to T cell recruitment (37, 41, 42). The corresponding chemokine receptor CXCR3 was expressed by Clone 4-GFP CD8^+^ T cells infiltrating the PA and to a much lower extent by HNT-CFP CD4^+^ T cells (Figure S2A in Supplementary Material). To determine whether signaling through this axis was involved in T cell migration in the PA, we treated transferred mice with anti-CXCR3 mAb 1 h prior *in vivo* imaging. We found that this treatment had minor effects on T cell average velocities in the exocrine tissue (Figure 2F) without changing the nature of migration statistics (Figure 2G). By contrast, treatment with anti-CXCR3 mAb increased Clone 4-GFP CD8^+^ T cell and HNT-CFP CD4^+^ T cell motility in islets (Figure 2H) and significantly reduced Clone 4-GFP CD8^+^ T cell recruitment into the PA (Figure S2B in Supplementary Material). Reduced effects on CD4^+^ T cells compared to CD8^+^ T cells may be explained by a lower receptor expression on the former cells (Figure S2 in Supplementary Material). Thus, while CXCR3 has minor involvement in CD8^+^ and CD4^+^ T cell migratory pattern in the exocrine tissue, it actively participates in CD8^+^ T cell recruitment and downregulates T cell velocity in islets, presumably to promote the confinement of effector cells and local accumulation at sites of chemoattractant production (35) and/or cognate antigen presence.

### Blood Vessels and ECM Fibers Provide a Scaffold for T Cell-Directed Motility in the Exocrine Tissue *In Vivo*

**Figure 3:**
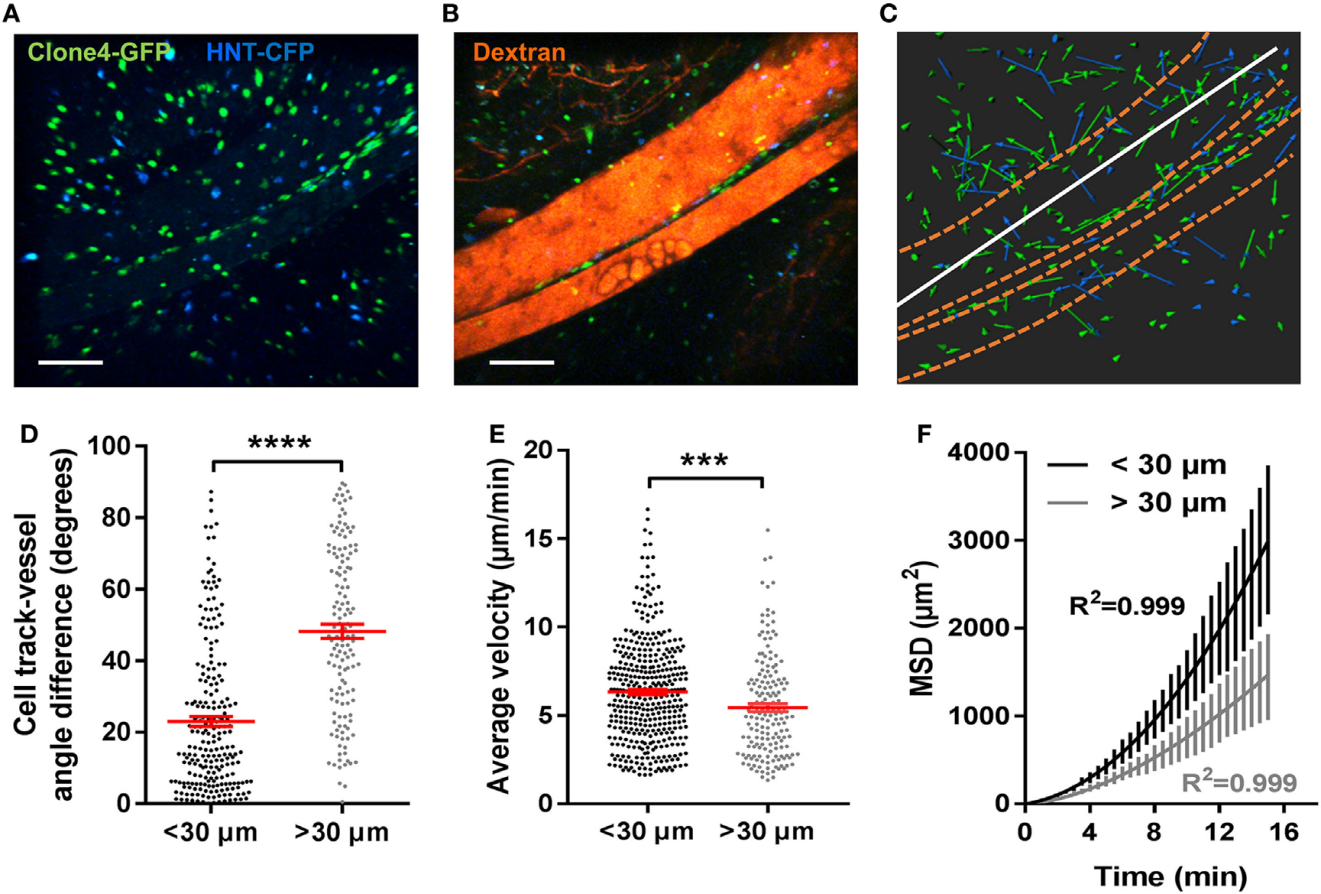
Blood vessels in the exocrine tissue contribute to effector T cells-directed mode of motility. Irradiated InsHA-mCherry mice adoptively transferred with Clone 4-GFP CD8^+^ and HNT-CFP CD4^+^ T cells were subjected to intra-vital microscopy on day 8. **(A)** Still images from a movie at day 8 post transfer (scale: 100- and 300-µm *Z*-projections) **(B)** Still image from the movie in (A) post-injection of 150-kDa dextran-rhodamine (scale: 100- and 300-µm *Z*-projection). **(C)** *XY* projections of track displacement vectors of T cells in the movie depicted in **(A)**. Blue and green tracks correspond to HNT-CFP and Clone 4-GFP T cells, respectively. Dashed orange lines outline large vessels and white lines indicate axis used to calculate angles between displacement vectors and vessel positions. **(D)** Angle differences between displacement vectors of T cells close to vessels (*n* = 3 movies from 3 mice) are lower than that of T cells away (>30 µm) from vessels (Mann–Whitney). **(E)** The average velocities of T cells close or away from vessels (>30 µm) (*n* = 3 mice/condition; 1 movie/mouse, Mann–Whitney). Dots correspond to individual T cells. **(F)** MSD of T cells close to (<30 µm) vessels as a function of time, best fitted with a directed model of migration, while other T cells follow a Lévy walk (>30 µm from vessels). Bars correspond to SEM (*n* = 4 mice, 1 movie/mouse).

Diabetogenic T cells have been shown to extravasate and infiltrate the PA both within islets (24) and from post-capillary venules in the exocrine tissue (14). In accordance with this, early infiltration events here were limited to islets and perivascular areas (Figure S3A in Supplementary Material). As infiltration progressed, large accumulations of effector CD4^+^ and CD8^+^ T cells could be observed within islets and at the level of endomucin-expressing pancreatic venules on fixed PA sections (Figure S3B in Supplementary Material) and *in vivo* (Figure S3C in Supplementary Material). Along large vessels (>100 µm in diameter), T cells displayed linear tracks (Figures 3A–C). To quantify alignment between T cell tracks and vessels, angle differences between track displacement vectors and vessel direction (1) (white lines, Figure 3C) were measured for T cells close to or away from vessels (<30 µm or >30 µm) (Figure 3D). Compared to other T cells in the imaging field, T cells in close proximity to vessels presented lower angle differences with vessel orientation, increased velocity, and fully ballistic motility (Figures 3D–F). Thus, the vascular structure strongly influences all parameters of T cell migration in the PA. This is consistent with what was observed in the islet periphery in the NOD mouse model of T1D (19).

**Figure 4:**
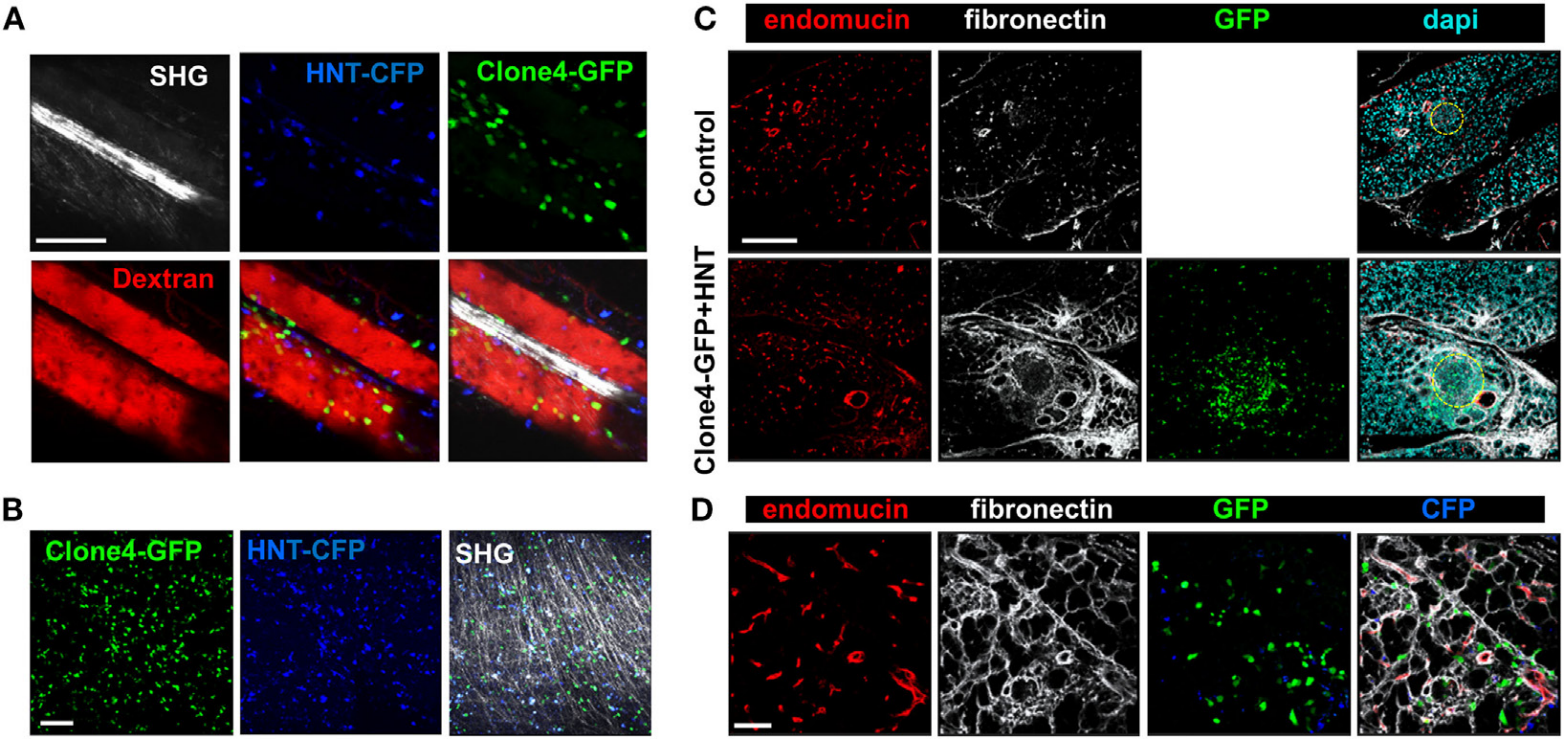
Effector T cells migrate along ECM fibers, which accumulate at infiltration sites. Irradiated InsHA-mCherry mice adoptively transferred with Clone 4-GFP CD8^+^ and HNT-CFP CD4^+^ T cells were subjected to intra-vital microscopy on day 8. **(A)** Still images from a movie at day 8 post transfer (scale: 100- and 87-µm *Z*-projection) (see also Videos S3 in Supplementary Material). SHG, second harmonic generation; red, rhodamine-dextran. **(B)** Still images from a movie at day 8 post transfer (scale: 100- and 100-µm *Z*-projection) (see also Videos S4 in Supplementary Material). SHG, second harmonic. **(C)** Representative confocal images of PA of a control irradiated InsHA-mCherry mouse, or post-transfer of T cells (scale: 200 µm, *Z*-projection of 20 µm). Islets are circled. **(D)** Representative confocal images of exocrine tissue at day 8 post transfer (scale: 50 µm, *Z*-projection of 4 µm), showing transferred T cells in close apposition to fibronectin fibers.

Since different components of the ECM have been involved in guiding effector T cells migration through ligand–receptor interactions (1, 15, 16), and vessels are usually lined with a dense accumulation of ECM fibers, we analyzed T cell motility *in vivo* on ECM fibers visualized by SHG. We found the T cells were able to follow dense ECM bundles between vessels (Figure 4A; Video S3 in Supplementary Material). In addition, ECM fibers could be observed in the infiltrated exocrine tissue, although SHG was limited to the tissue surface (Figure 4B; Video S4 in Supplementary Material). Because ECM composition may be modulated by inflammation (25), we investigated whether PA infiltration was accompanied by changes in the ECM. Fibronectin, a key component of the ECM and a major substrate for matrix-binding integrins (1), could be evidenced in the PA of non-treated control mice and localized to the perivascular space, as well as the interstitial tissue around cells in the exocrine PA (Figure 4C). At day 8 post T cell transfer, we found an increase in fibronectin deposition at T cell infiltration sites (Figure 4C). This was also true for other components of the ECM, such as collagen I (Figure S4A in Supplementary Material). Importantly, the assessment of Clone4-GFP and HNT-GFP T cells localization revealed a generalized close apposition to fibronectin fibers in pre-diabetic mice (Figure 4D). Other changes in the micro-environment accompanying T cell infiltration, and locally correlated with fibronectin accumulation, included important APC recruitment, as evidenced by dense CD11c and F4/80 labeling around and within islets and around blood vessels (Figure S4B in Supplementary Material). Recruited T cells therefore migrate around a restructured scaffold of ECM fibers and leukocytes.

### β1 Integrin Blockade Alters Directed Effector T Cell Migration in the PA and Impairs Their Effector Phenotype

**Figure 5:**
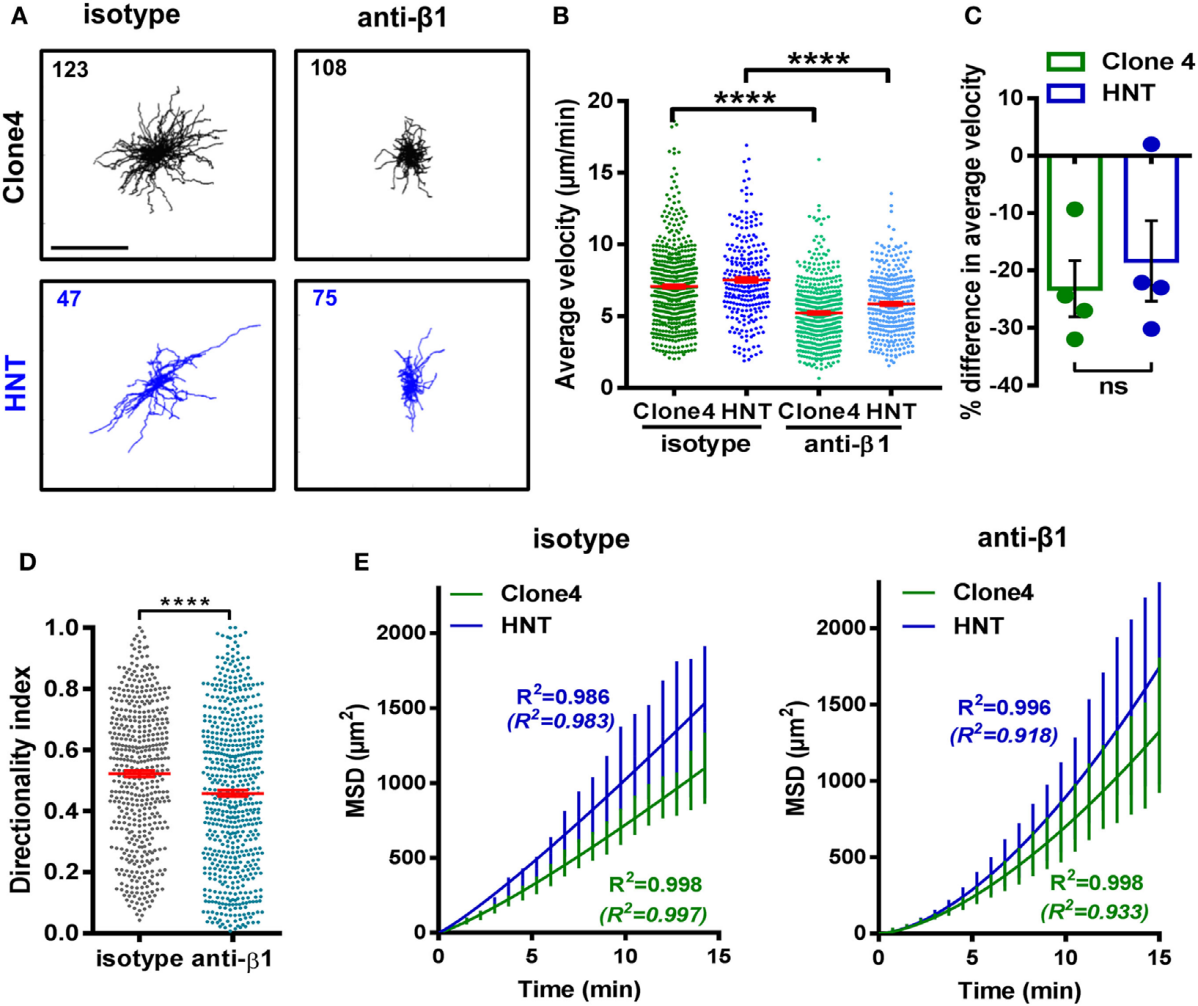
β_1_ integrin-dependent interactions between T cells and the ECM shape T cell motility. Irradiated InsHA-mCherry mice transferred with Clone 4-GFP and HNT-CFP CD4^+^ T cells were subjected to intra-vital microscopy on day 8. **(A)** *XY* projections of T cell tracks over 15.2 min, 35 min after control IgG or anti-β_1_ integrin injection (scale: 100 µm) (see also Video S5 in Supplementary Material). Values indicate the number of tracks in movies. **(B)** The average velocity of T cells in the exocrine tissue of isotype and anti-β_1_ integrin-treated animals (*n* = 3 mice/condition; 1–2 movies/mouse; one-way ANOVA). Dots correspond to individual cells. **(C)** Percentage difference in the average velocity between T cells in the exocrine tissue of isotype and anti-β_1_ integrin-treated animals. Dots correspond to individual movies (*n* = 3 mice/condition; 1–2 movies/mouse; *P* = 0.48, Mann–Whitney). **(D)** Directionality index of T cells in exocrine tissue (*n* = 3 mice/condition; 1–2 movies/mouse, Mann–Whitney). **(E)** MSD of T cells as a function time in the exocrine tissue of isotype and anti-β_1_ integrin-treated animals were both best fitted with a model of Lévy-type super-diffusive migration (solid lines). Between brackets are *R*^2^-values of fit for Brownian random motility. Bars correspond to SEM (*n* = 3 mice/CD8^+^ condition; 1–2 movies/mouse).

RGD-binding integrins are known receptors for ECM proteins and in particular for fibronectin. Therefore, we assessed the expression of those that have been reported to be more frequently present on diabetogenic T cells (1) in the infiltrating effector T cells of our model. We found that the vast majority of both Clone 4-GFP CD8^+^ and HNT CD4^+^ T cells expressed high levels of β_1_ and α_V_ integrins (Figures S5A,B in Supplementary Material). We hypothesized that integrins could be involved in guiding effector T cell motility in the PA. We tested this hypothesis by injecting a blocking anti-β_1_ integrin mAb. To determine optimal imaging time post injection and control for potential micro-anatomical changes between different imaging fields, we injected mAbs through a catheter inserted in the jugular vein and monitored the average T cell motility in the same field pre- and post injection. We found that a maximum effect was reached by 35–50 min post injection using blocking anti-β_1_ integrin mAb while isotype control mAb had no effect on T cell velocity (Figure S5C in Supplementary Material). From this time point, the average velocities of both Clone 4 and HNT T cells were significantly reduced compared to isotype control antibody-treated animals (~20%) (Figures 5A–C; Video S5 in Supplementary Material), as well as the directionality indexes of T cell tracks (Figure 5D). In addition, although T cell MSD versus time curves were still best fitted with the Lévy-type random model, curves tended to linearize and the fit for a Brownian-type random motility improved in treated animals (Figure 5E). To further assess the involvement of integrins in T cell motility in the PA, we treated animals prior to imaging (10 min) with a peptide containing the RGD peptidic motif. Since this sequence is recognized by β_1_ integrin on ECM fibers, treatment with RGD peptide broadly blocks a large fraction of matrix-binding integrins. We found that the average T cell velocity was decreased compared to reverse DGR peptide-treated animals (Figures S6A,B and Video S6 in Supplementary Material) and super-diffusive motility was practically lost (Figure S6C in Supplementary Material). This indicates that matrix-binding integrins contribute to T cell motility in the inflamed PA, although compensatory and/or additional mechanisms may exist (17, 20).

**Figure 6:**
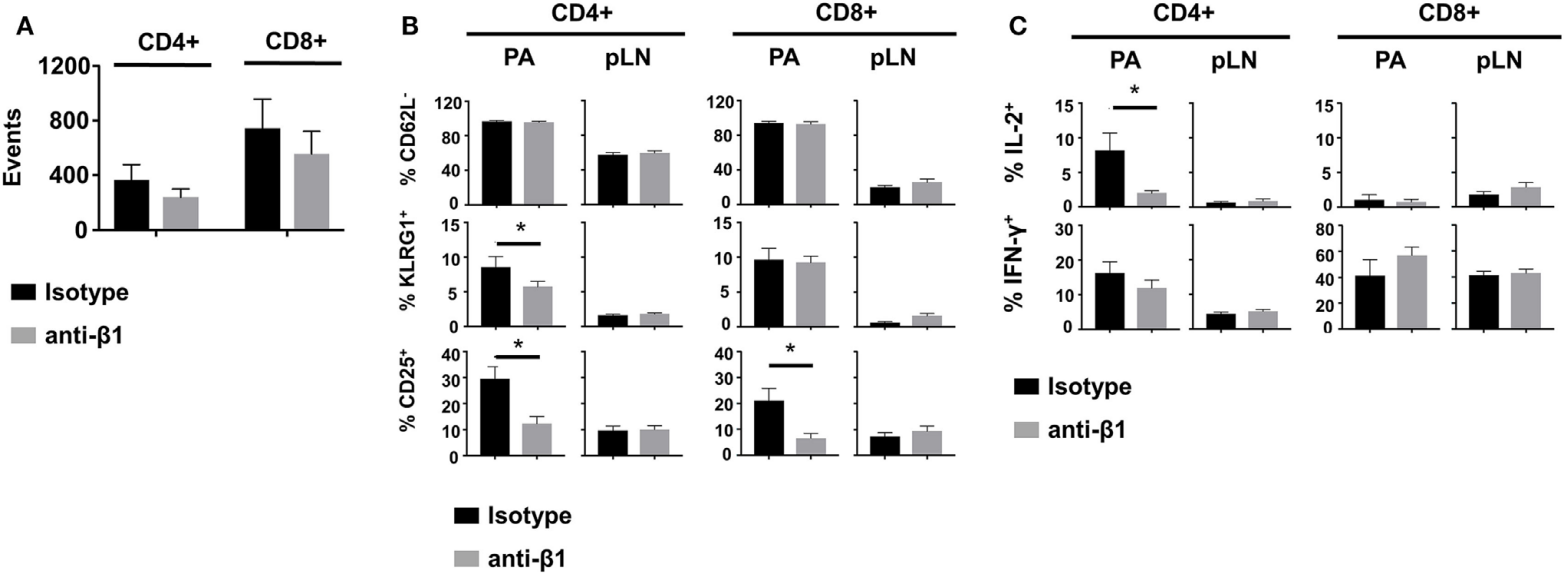
β_1_ integrin blockade alters diabetogenic T cell effector phenotype in the pancreas (PA). Irradiated InsHA-mCherry mice transferred with Clone 4-GFP CD8^+^ and HNT-CFP CD4^+^ T cells were treated with anti-β1 mAb or isotype control antibodies on days 8 and 9 after transfer. At day 10, donor T cells from pancreatic LN (pLN) and PA were analyzed by FACS gating on living CD8^+^ or CD4^+^ Thy1.1^+^ lymphocytes. **(A)** Donor T cells in the PA of treated mice. FACS event counts in the CD8^+^ or CD4^+^ Thy1.1^+^ gates from three independent experiments, represented as mean ± SEM (*n* = 8 mice, Mann–Whitney). **(B)** Donor T cell expression of CD25, CD62L, and KLRG1. Percentages of indicated subpopulations in the CD8^+^ Thy1.1^+^ or CD4^+^ Thy1.1^+^ gates from three independent experiments, represented as mean ± SEM (*n* = 8 mice, Mann–Whitney). **(C)** Intracellular cytokine measurement in donor T cells. Percentages of IL-2^+^ or IFNγ^+^ cells in the CD8^+^ Thy1.1^+^ or CD4^+^ Thy1.1^+^ gates from two independent experiments, represented as mean ± SEM (*n* = 6 mice, Mann–Whitney).

Finally, we tested whether impaired effector T cell motility induced by β_1_ integrin blockade could affect functionality. Mice were treated with anti-β_1_ integrin mAb at a time at which T cells had already started infiltrating the PA (days 8 and 9 after transfer). At day 10, equal numbers of infiltrating Clone 4-GFP CD8^+^ and HNT-CFP CD4^+^ T cells were detected in the PA of treated compared to isotype control mice (Figure 6A). Moreover, the phenotype and cytokine secretion potential of both donor CD8^+^ and CD4^+^ T cells were indistinguishable in the draining LNs of the PA (Figures 6B,C). These results indicate that treatment at this time point did not prevent further recruitment of effector cells into the PA nor activation of effector cells in the pancreatic LNs. By contrast, PA infiltrating HNT-CFP CD4^+^ T cells from treated mice displayed a significant reduction in the expression of key effector markers such as KLRG1 and CD25 (Figure 6B). In addition, these cells had lost the potential to secrete IL-2, an important effector cytokine in our model (Figure 6C) (26, 27). This was, however, not accompanied by a statistically significant decrease in IFNγ production. Finally, although the effector potential of Clone 4-GFP CD8^+^ T cells remained unaltered, a marked reduction of the expression of CD25 was observed in treated mice, likely as a result of the decreased IL-2 secretion by helper CD4^+^ T cells (Figure 6C). Collectively, our data indicate that altered motility of diabetogenic T cells in the PA results in deceased effector functions *in situ*.

## Discussion

T cell migratory behavior stems from the need to search for their cognate antigen and plays a crucial role in antigen clearance. In various peripheral tissues (brain, liver, gut, PA), T cell migratory behavior has been described as a super-diffusive random walk or Lévy walk (4, 14, 40), characterized by steps of directed migration in random directions interleaved by pauses (43), to optimize rare target encounter. However, mechanisms governing T cell migration are context-dependent, and leukocytes are able to switch migratory modes along with changing environmental conditions (1). This prevents the definition of a generalized model for T cell interstitial migration in inflamed peripheral tissues. In addition, mechanisms governing lymphocyte dynamics are intimately linked to the maintenance of T cell effector function (1). While effector T cells need to reach dispersed target islets in the PA during autoimmune diabetes, mechanisms governing their motility remained unclear. Using 2-photon microscopy *in vivo* to visualize TCR transgenic HA-specific CD8^+^ and CD4^+^ T cells in the PA of mice expressing HA in beta cells, we found that both T cell types followed a super-diffusive Lévy-type mode of migration in the exocrine tissue without a preferred concerted orientation. By contrast, the islet environment restrained T cell trafficking through a mechanism involving CXCR3 chemokine receptor. T cell infiltration induced local fibrosis, marked by fibronectin deposition. Both CD8^+^ and CD4^+^ T cells were in close apposition to vessels and fibronectin fibers, which provided adhesive guidance and contributed to the super-diffusive migration in the exocrine PA through, at least partially, a β_1_ integrin-dependent mechanism. Finally, β_1_ integrin-dependent T cell–ECM interactions contributed to the maintenance of T cell effector function in the PA.

The InsHA transgenic mouse model provides a powerful tool to rapidly and synchronously induce diabetes and study antigen-specific T cell behavior in the PA during autoimmune diabetes onset (27). Although a high pathogenic T cell frequency resulting from the transfer of HA-specific TCR transgenic T cells may not entirely recapitulate the pathogenesis of the spontaneous slow-progressing disease, results obtained here share characteristics with those found analyzing endogenous T cell repertoire in NOD mice, in which spontaneous autoimmune diabetes develops (19). Common features include abundant T cell interactions with APCs and T cell migration guided by blood vessels, supporting the physiological relevance of our results.

In search for their cognate antigen, T cells can follow a “Brownian” random walk mode of migration in the periphery, including in the PA (6, 14). Here, however, T cells followed a super-diffusive, almost ballistic, mode of migration (4), which arguably constitutes the most efficient strategy of random search processes (44). The different migration pattern observed in the exocrine tissue by Coppieters et al. may rise from model-specific differences (autoimmune diabetes was induced using viral infection) and/or different length of movie duration to analyze T cell MSD (<7 min versus 12–16 min here) (14). Strikingly, T cells did not collectively migrate in a particular direction as no overall orientation bias of T cell tracks was observed, including toward islets, although these are major sources of chemokines (37). We found that, unlike in LN (45), Gαi-coupled receptors involved in chemokine signaling, such as CXCR3 receptors, were not central in shaping T cell motility in the exocrine PA. CXCR3 blockade slightly reduced T cell velocity without affecting migration mode, as reported previously in the brain (43). On the local scale, T cells were able to follow each other for extended periods of time (>5 min, 1–2 events per 15-min movie, data not shown), suggesting that they may follow paths of least resistance. Alternatively, like recently described for neutrophils (46), T cells may be able to deposit chemokine trails that other T cells may be able to respond to, although this remains unclear.

Although the original assumption was that large-scale diffusive chemokine gradients would provide cues for directed motility, experimental evidence of collective T cell migration toward sources of high chemokine production is scarce. By contrast, chemotactic cues are able to modulate T cell trajectories in different ways, such as through modulation of T cell retention/arrest rather than directionality (35). In accordance with this, a large accumulation of T cells was observed in islets and CXCR3 blockade increased T cell velocity in islets, as beta cells are the main source of CXCL9/10 in the PA (37). The chemokine-rich environment of islets therefore promotes a downregulation of T cell velocity to accumulate and confine effector cells at target sites, rather than attract distant T cells. The dense accumulation of T cells at the level of post-capillary venules in the exocrine tissue could be explained by the described vascular leakiness (14) and the presence along vessels of CD11c^+^ and F4/80^+^ cells, which are well-known chemokine sources that could favor confinement of T cells.

Similar to what was described in the inflamed skin (1), the vascular tree provided a scaffold for T cell migration in the PA and strongly contributed to the directional motility *in vivo*. In addition, HA-specific CD8^+^ and CD4^+^ T cell infiltration induced ECM remodeling, likely mediated by recruited macrophages (47). This remodeling included fibronectin accumulation, a major substrate for matrix-binding integrins (1). The fact that anti-β1 integrin mAb treatment affected both velocity and directionality of T cells indicates that lymphocytes do not only align along paths of least resistance in the PA, but that fibronectin fibers also provide adhesive guidance. Effects observed were in line with previous studies of integrin blockade on T cell motility (48). By contrast, with full matrix-binding integrin-dependency described in the inflamed skin (1), our results suggest the implication of complementary mechanisms of migration for T cells in the PA. Once the described chemokine-dependent upregulation of endothelial cell-binding integrin molecules allowing T cell entry at peripheral sites has been achieved (49), infiltrated T cell directional migration in the PA is mostly independent of CXCR3-mediated chemotactic signals. The remaining migration detected in the presence of β_1_ integrin-blocking antibody may stem from T cells’ intrinsic capacity to maintain a directed motion (50), other GPCR-mediated chemokine signaling (51), and/or other receptor–ligand interactions, although this remains to be clarified.

Finally, integrin β1 blockade at a time when diabetogenic T cells had already infiltrated the PA resulted in a decline of CD4^+^ T cell effector function, marked by a loss of potential to secrete the effector cytokine IL-2. A possible explanation may be that impairing ECM-guided motility would alter effector CD4^+^ T cell/APC interactions resulting in a decrease in effector function. Indeed, diabetogenic T cells, including HNT CD4^+^ T cells, require antigen-mediated contacts with APCs in the PA to retain effector phenotype and functionality over time (27, 52). In contrast with effects on IL-2 secretion, IFNγ production, also subject to signals triggered by T cell/APC interactions, was only marginally affected. An alternative/complementary explanation may be that integrin signaling triggered by direct interaction with ECM fibers may also be required for the maintenance of effector functions in the PA. In particular, integrin β1 signaling has been shown to act as a co-stimulatory signal for IL-2 secretion by CD4^+^ T cells (53–55). Thus, such a mechanism could contribute to the differential effect of integrin β1 blockade on IL-2 versus IFNγ production, although this remains to be investigated.

In summary, we show that during autoimmune insult to the PA, islet-antigen-specific T cells display super-diffusive motility in the exocrine tissue, implicating β_1_ integrin-dependent T cell–ECM fiber interactions contributing to the optimization of islet encounter and maintenance of effector functions, and that the islet chemokine-rich environment promotes the confinement of effector T cells, rather than their recruitment. We thus reveal a role for matrix-binding integrins in the PA that may have important implications for the design of new therapeutic strategies against T1D.

## Ethics Statement

Animal studies were conducted according to the European guidelines for animal welfare (2010/63/EU). Protocols were approved by the Institutional Animal Care and Use Committee (CEEA-LR-1190 and -12163) and the French Ministry of Agriculture (APAFIS#3874).

## Author Contributions

GE-C, CS, JH, and MS designed experiments; GE-C, CS, AM, and MS performed experiments; GE-C, CS, PF, JH, and MS analyzed data; PM, JH, and MS wrote the manuscript.

## Conflict of Interest Statement

The authors declare that the research was conducted in the absence of any commercial or financial relationships that could be construed as a potential conflict of interest.

## Supplementary Material

Video S1 Intra-vital imaging of islet-antigen-specific CD4^+^ and CD8^+^ T cells in the pancreas (PA). *In vivo* recording of T cell motility around an infiltrated islet in the PA of an InsHA-mCherry mouse transferred with HNT-CFP CD4^+^ and Clone 4-GFP CD8^+^ T cells at day 8 post transfer. Movie rate: 4 frames/s. Total elapsed time: 15 min. Green, GFP; blue, CFP; red, mCherry. Image size: 610 μm× 610 μm, 208 μm *Z*-projection.

Video S2 Super-diffusive motility of T cells in the exocrine tissue. *In vivo* recording of T cells displaying directed motility in the pancreas of an InsHA-mCherry mouse transferred with HNT-CFP CD4^+^ and Clone 4-GFP CD8^+^ T cells at day 8 post transfer. Movie rate: 4 frames/s. Total elapsed time: 19 min. Green, GFP; blue, CFP; red, mCherry. Image size: 650 μm× 650 μm, 200 μm *Z*-projection.

Video S3 Migration of T cells along ECM fibers and blood vessels. *In vivo* recording of T cells migrating along second harmonic (SHG) signal generated by ECM fibers along blood vessels in the pancreas of an InsHA-mCherry mouse at day 8 post transfer of HA-specific T cells. The two panels depict the same movie, with SHG signal turned off on the right to be able to visualize T cells along SHG. Examples of T cells following ECM fibers are circled. Lines mark the intervascular space rich in ECM fibers along which T cells are moving directionally. Movie rate: 8 frames/s. Total elapsed time: 21 min. Green, GFP; blue, CFP; white, SHG. Image size: 280 μm× 280 μm, 87 μm *Z*-projection.

Video S4 Organized ECM fibers in the infiltrated exocrine tissue. *In vivo* recording of T cell migration within the mesh of second harmonic (SHG) signal generated by ECM fibers in the exocrine tissue of the pancreas of an InsHA-mCherry mouse at day 8 post transfer of HA-specific T cells. Movie rate: 8 frames/s. Total elapsed time: 4.5 min. Green, GFP; blue, CFP; white, SHG. Image size: 620 μm× 620 μm, 100 μm *Z*-projection.

Video S5 T cells motility is altered by blocking anti-β1 integrin mAb. *In vivo* recordings showing T cell motility 35–40 following i.v. injection of isotype control antibody (left) and anti-β1 integrin antibody (right) (100 μg), in the pancreas of InsHA-mCherry mice transferred with HNT-CFP CD4^+^ and Clone 4-GFP CD8^+^ T cells, at day 8 post transfer. Movie rates: 8 frames/s. Total elapsed time: 10 min for both movies. Green, GFP; blue, CFP. Image size: 600 μm× 600 μm, 180 μm *Z*-projection.

Video S6 T cells motility is altered by blocking RGD peptide. *In vivo* recording showing T cell motility 10–50 min following i.v. injection of DGR peptide (left) or RGD peptide (right) (500 μg/mouse) in the pancreas of an InsHA-mCherry mouse at day 8 post transfer of HA-specific T cells. Movie rate: 4 frames/s. Total elapsed time: 23 min for both movies. Green, GFP; blue, CFP. Image size: 610 μm× 610 μm, 160 μm *Z*-projection.
