# Supplementary material for: Integrin β1 Optimizes Diabetogenic T Cell Migration and Function in the Pancreas
Source: Front Immunol. 2018 May 31;9:1156. doi: 10.3389/fimmu.2018.01156 (PMC5990596; doi:10.3389/fimmu.2018.01156)
Supplement: Supplementary file 7 [file data_sheet_1.PDF]

**SUPPLEMENTAL INFORMATION**

**SUPPLEMENTAL FIGURES**

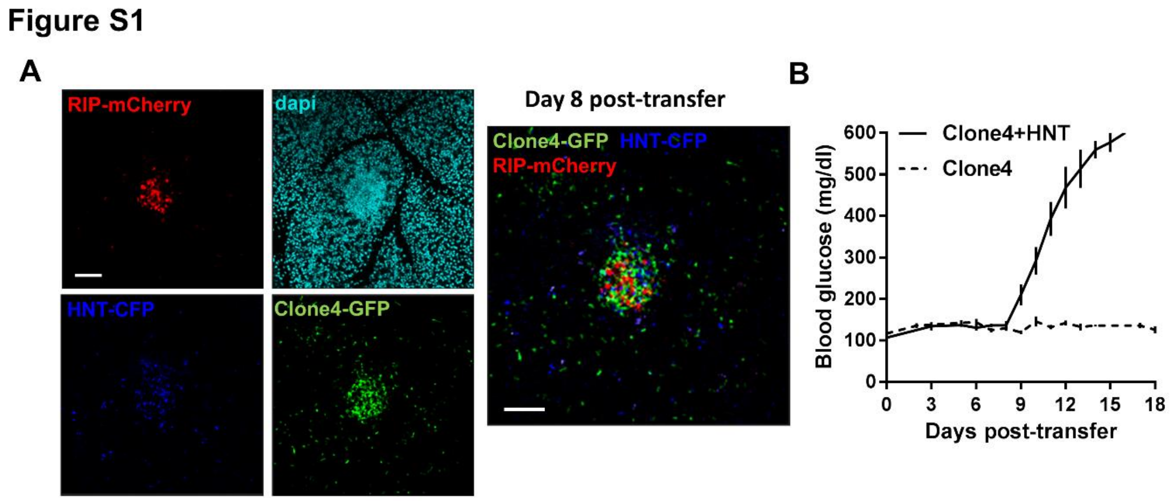


**Figure S1. Murine model of synchronized autoimmune attack of islets by islet-antigen specific T cells. A)** Irradiated InsHA-mCherry mice adoptively transferred with Clone 4-GFP $CD8^+$ and HNT-CFP $CD4^+$ T cells were analyzed on day 8 after transfer. Representative confocal images of a pancreas showing accumulation of transferred cells (green: GFP, blue: CFP) within islets (red) and in the exocrine tissue (scale: 100 µm, Z-projection of 24 µm). **B)** Blood glucose levels of irradiated InsHA-mCherry mice as function of days post-transfer of Clone 4-GFP $CD8^+$ alone, or Clone 4-GFP $CD8^+$ and HNT-CFP $CD4^+$ T cells (n = 16-28 mice/condition).

Figure S2

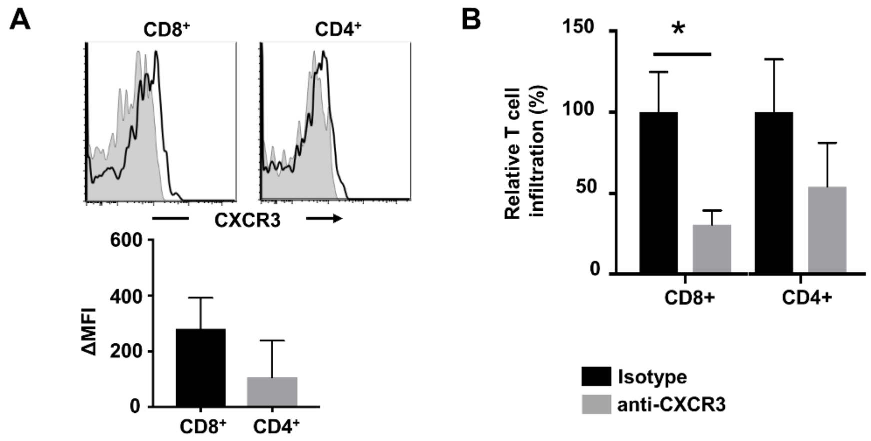


**Figure S2. Expression of CXCR3 receptor on T cells and effect of anti-CXCR3 mAb treatment on T cell recruitment to the pancreas.** Irradiated InsHA-mCherry mice were adoptively transferred with Clone 4-GFP CD8$^+$ and HNT-CFP CD4$^+$ T cells. **A**) Pancreas infiltrating donor T cells were analyzed at day 9 for expression of CXCR3 chemokine receptor by FACS gating on living CD8$^+$ Thy1.1$^+$ or CD4$^+$ Thy1.1$^+$ lymphocytes. Histograms depicts data from a representative mouse with shaded isotype control. Graphs represent mean ± SEM of differences in Maximum Fluorescence Intensity (ΔMFI) with isotype controls from two independent experiments (n = 6 mice). **B)** Host mice were treated with either anti-CXCR3 or isotype control antibody on days 8 and 9 after transfer. At day 10, donor T cell infiltration in pancreas was analyzed by FACS gating on living CD8$^+$ Thy1.1$^+$ or CD4$^+$ Thy1.1$^+$ lymphocytes. Graphs represent mean ± SEM (n = 5 mice) of percentage of FACS events present in treated mice relative to control mice from 2 independent experiments.

**Figure S3**

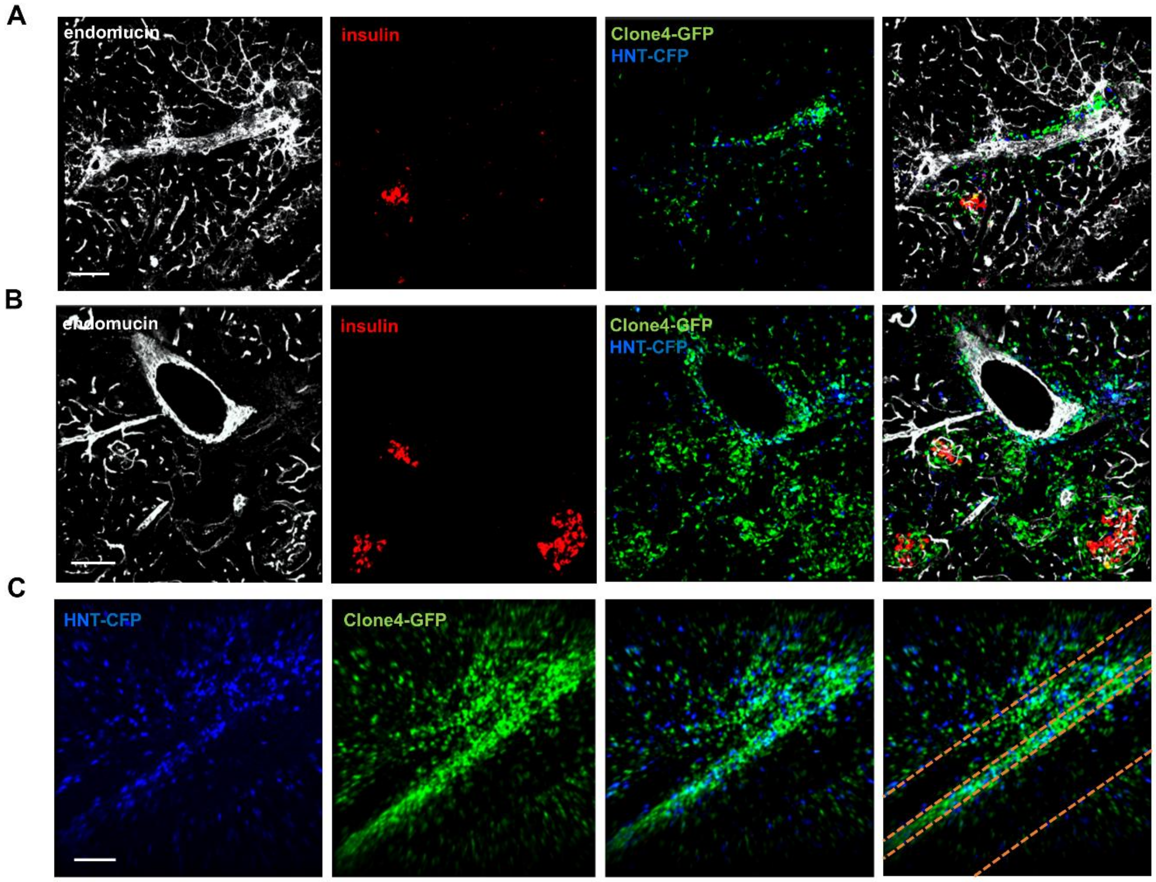


**Figure S3. Effector T cells accumulate within islets and around venules.** Irradiated InsHA-mCherry mice adoptively transferred with Clone 4-GFP $CD8^+$ and HNT-CFP $CD4^+$ T cells were subjected to intra-vital microscopy on day 8. **A-B)** Representative confocal images of a pancreas showing accumulation of transferred cells (green: GFP, blue: CFP) within islets (red) and around endomucin$^+$ venules (white) (scale: 100 µm, Z-projection of 20 µm). **C)** Still images acquired *in vivo* showing accumulation of T cells along vessels (orange dashed lines) (Scale: 100 µm, 200 µm Z-projection).

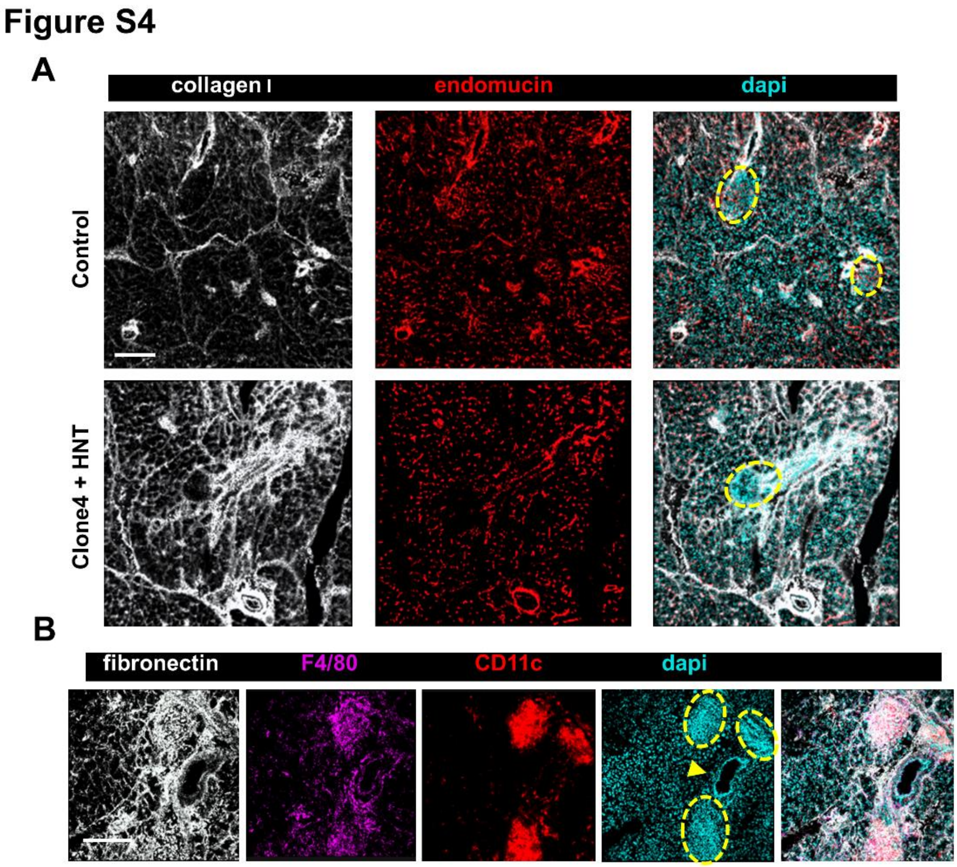


**Figure S4. Localized micro-environmental changes accompanying T cell infiltration include ECM alterations and APCs recruitment. A)** Representative confocal images of pancreas of an untransferred irradiated mouse, and of an InsHA-mCherry mouse at day 8 post T cell-transfer (scale: 200 µm, Z-projection of 20 µm). **B)** Representative confocal images of pancreas at day 8 post-transfer (scale 200 µm, Z-projection of 9 µm). Islets are circled. Yellow arrow indicates the position of a blood vessel, surrounded by APCs.

Figure S5

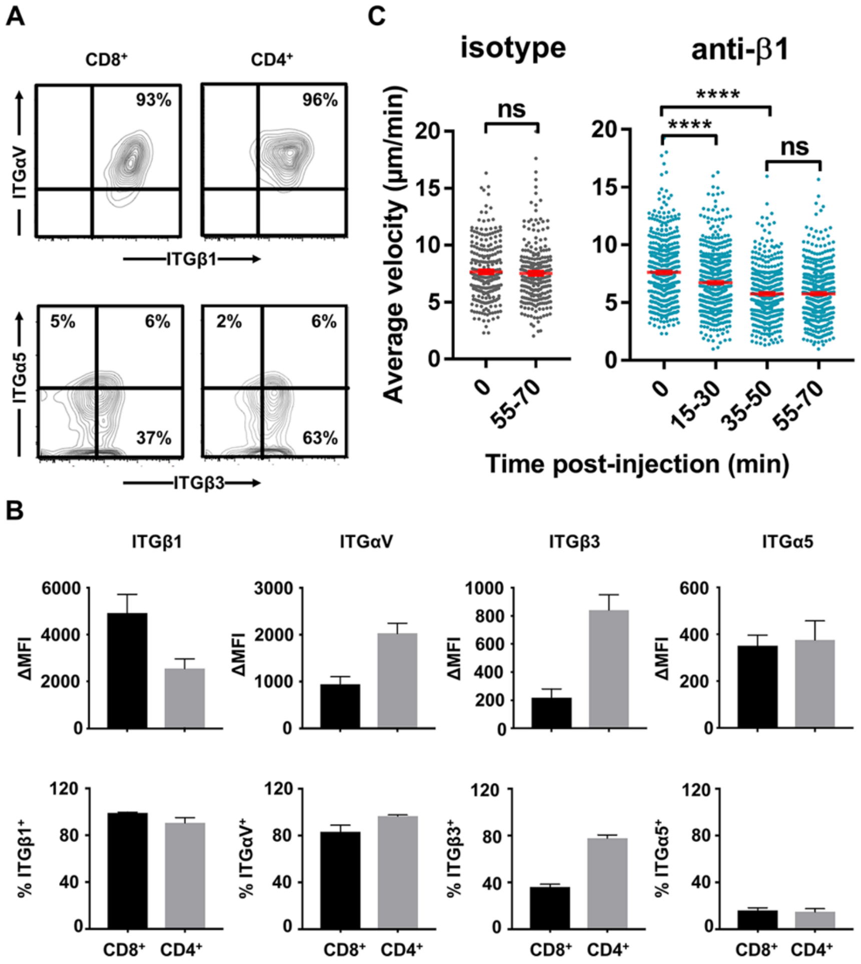


**Figure S5. Integrin expression in islet-antigen specific T cells infiltrating the pancreas.** Irradiated InsHA-mCherry mice were adoptively transferred with Clone 4-GFP CD8$^{+}$ and HNT-CFP CD4+ T cells. At day 9 after transfer, pancreas infiltrating donor T cells were analyzed for the expression of the indicated integrins by FACS gating on living CD8$^{+}$ Thy1.1$^{+}$ or CD4$^{+}$ Thy1.1$^{+}$ lymphocytes. ITG: integrin. **A)** Plots depict data from a representative individual mouse with quadrants positioned according to isotype controls. **B)** Graphs represent mean ± SEM of differences in Maximum Fuorescence Intensity (ΔMFI) (top panels) or percentages of the indicated subpopulations (bottom panels) compared to the isotype control from two independent experiments (n = 6 mice). **C)** Irradiated InsHA-mCherry mice transferred with Clone 4-GFP and HNT-CFP T cells were subjected to intra-vital microscopy on day 8. Monitoring of average T cell velocity over time in the same imaging field following isotype (left) and anti-$\beta_1$ integrin (right) mAb injection (n = 2 mice/condition; 1 movie/mouse/time point; One-way Anova). Values represent mean ± SEM.

Figure S6

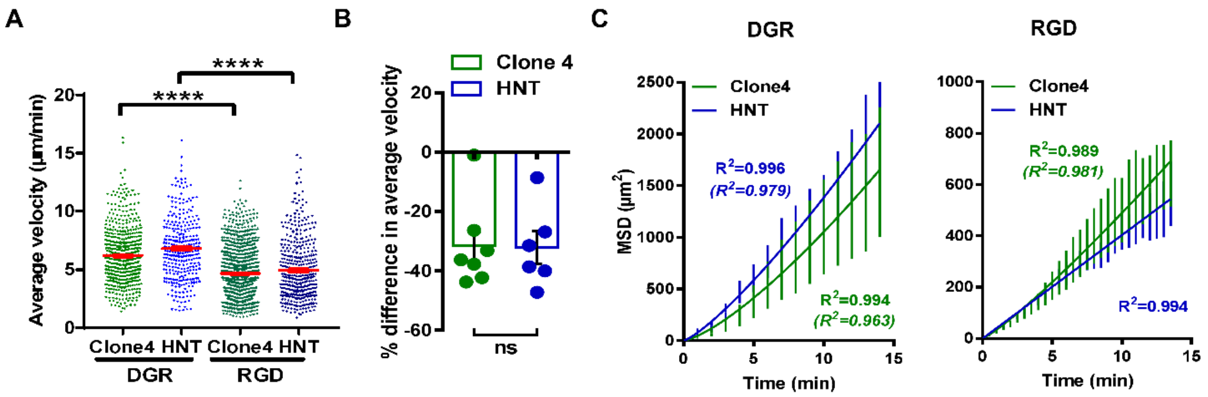


**Figure S6. RGD peptide impinges on T cell velocity and super-diffusive migration.** Irradiated InsHA-mCherry mice adoptively transferred with Clone 4-GFP CD8$^+$ and HNT-CFP CD4$^+$ T cells were subjected to intra-vital microscopy on day 8. **A)** Average velocity of T cells in the exocrine tissue of RGD or reverse DGR peptide-treated animals (n = 4-5 mice/condition; 1-3 movies/mouse; Mann-Whitney). **B)** Percentage difference in average velocity between T cells in the exocrine tissue of RGD or reverse DGR peptide-treated animals. Dots correspond to individual movies (n = 4-5 mice/condition; 1-3 movies/mouse; P = 0.94, Mann-Whitney). See also Video S6. **C)** MSD of T cells as function of time in the exocrine tissue of DGR peptide-treated animals were best fitted with a model of Lévy-walk (left panel), while MSD of CD8$^+$ and CD4$^+$ T cells as function time in the exocrine tissue of RGD peptide-treated animals were best fitted with a model of Lévy-walk and Brownian-type random motility (linear regression), respectively (solid lines). Bars correspond to SEM of pooled data (n = 3 mice/condition; 1-3 movies/mouse). Between brackets are $R^2$ values of fit for Brownian random motility. Values represent mean ± SEM.

## SUPPLEMENTAL VIDEOS

**Video S1. Intra-vital imaging of islet antigen-specific CD4$^+$ and CD8$^+$ T cells in the pancreas.** *In vivo* recording of T cell motility around an infiltrated islet in the pancreas of an InsHA-mCherry mouse transferred with HNT-CFP CD4$^+$ and Clone 4-GFP CD8$^+$ T cells at day 8 post-transfer. Movie rate: 4 frames/s. Total elapsed time: 15 min. Green: GFP, blue: CFP, red: mCherry. Image size: 610 × 610 µm, 208 µm z-projection.

**Video S2. Super-diffusive motility of T cells in the exocrine tissue.** *In vivo* recording of T cells displaying directed motility in a pancreas of an InsHA-mCherry mouse transferred with HNT-CFP CD4$^+$ and Clone 4-GFP CD8$^+$ T cells at day 8 post-transfer. Movie rate: 4 frames/s. Total elapsed time: 19 min. Green: GFP, blue: CFP, red: mCherry. Image size: 650 × 650 µm, 200 µm z-projection.

**Video S3. Migration of T cells along ECM-fibers and blood vessels.** *In vivo* recording of T cells migrating along second harmonic (SHG) signal generated by ECM-fibers along blood vessels in a pancreas of an InsHA-mCherry mouse at day 8 post-transfer of HA-specific T cells. The two panels depict the same movie, with SHG signal turned off on the right to be able to visualized T cells along SHG. Examples of T cells following ECM-fibers are circled. Lines mark the intervascular space rich in ECM-fibers along which T cells move directionally. Movie rate: 8 frames/s. Total elapsed time: 21 min. Green: GFP, blue: CFP, white: SHG. Image size: 280 × 280 µm, 87 µm z-projection.

**Video S4. Organized ECM-fibers in the infiltrated exocrine tissue.** *In vivo* recording of T cells migration within the mesh of second harmonic (SHG) signal generated by ECM-fibers in the exocrine tissue of a pancreas of an InsHA-mCherry mouse at day 8 post-transfer of HA-

specific T cells. Movie rate: 8 frames/s. Total elapsed time: 4.5 min. Green: GFP, blue: CFP, white: SHG. Image size: 620 × 620 µm, 100 µm z-projection.

**Video S5. T cells motility is altered by blocking anti-β1 integrin mAb.** *In vivo* recordings showing T cell motility 35-40 following i.v. injection of isotype control antibody (left) and anti-β1 integrin antibody (right) (100 µg), in the pancreas of InsHAmCherry mice transferred with HNT-CFP $CD4^+$ and Clone 4-GFP $CD8^+$ T cells, at day 8 post-transfer. Movie rates: 8 frames/s. Total elapsed time: 10 min for both movies. Green: GFP, blue: CFP. Image size: 600 × 600 µm, 180 µm z-projection.

**Video S6. T cells motility is altered by blocking RGD peptide.** *In vivo* recording showing T cell motility 10-50 min following i.v. injection of DGR peptide (left) or RGD peptide (right) (500 µg/mouse) in the pancreas of InsHAmCherry mouse at day 8 post-transfer of HA-specific T cells. Movie rate: 4 frames/s. Total elapsed time: 23 min for both movies. Green: GFP, blue: CFP. Image size: 610 × 610 µm, 160 µm z-projection.
